# Efficacy and Safety of Platelet-Rich Plasma (PRP) Intra-articular Injections in Hip Osteoarthritis: A Systematic Review of Randomized Clinical Trials

**DOI:** 10.7759/cureus.72057

**Published:** 2024-10-21

**Authors:** Abdullah N Almutairi, Mohammad S Alazzeh

**Affiliations:** 1 Orthopaedics, Al-Farwaniya Hospital, Kuwait Ministry of Health, Al-Farwaniyah, KWT; 2 Research, Tower Health, Phoenixville, USA

**Keywords:** coxarthrosis, femoroacetabular, hip, intra articular injection, oa, orthobiologic, osteoarthritis of the hip, platelet-rich plasma, prp

## Abstract

Hip osteoarthritis (OA) is a prevalent condition causing significant pain and disability. Platelet-rich plasma (PRP) intra-articular injections have emerged as a potential therapeutic option, but their efficacy is still debatable and their safety profile remains under-explored compared to standard treatments. This systematic review aims to evaluate the efficacy and safety of PRP injections in patients with hip OA by analyzing data from randomized clinical trials (RCTs). A comprehensive literature search was conducted in PubMed, Scopus, and the Virtual Health Library (VHL) until October 31, 2022, adhering to Preferred Reporting Items for Systematic reviews and Meta-Analyses (PRISMA) guidelines. Studies were included if they were RCTs assessing PRP injections for hip OA and reporting adverse events. Data extraction and methodological quality assessment were performed using the Cochrane Risk of Bias Tool (RoB 2 tool). Out of 188 identified studies, five met the inclusion criteria. The studies varied in sample size (43-111 patients) and PRP preparation methods (closed vs. open systems). All studies demonstrated significant pain reduction and functional improvement with PRP. No major adverse events were reported, indicating a favorable safety profile. Minor side effects were transient and resolved without further intervention. Methodological quality ranged from low to high risk of bias. In conclusion, PRP injections appear to be a safe and effective treatment option for managing hip OA, with favorable outcomes compared to hyaluronic acid. Further research is necessary to standardize PRP protocols and assess long-term safety and efficacy.

## Introduction and background

Hip osteoarthritis (OA) is a chronic degenerative joint disease characterized by the breakdown of cartilage, formation of osteophytes, and changes in the subchondral bone. It is a leading cause of pain and disability among adults, significantly impacting the quality of life and imposing substantial economic burdens on healthcare systems globally as reported by Bourne et al. and Cross et al. [1,2]. The pathophysiology of hip OA involves complex interactions among mechanical stress, biochemical mediators, and genetic factors, leading to progressive joint damage and inflammation [3].

Conventional treatments for hip OA include a combination of non-pharmacological and pharmacological strategies aimed at symptom relief and functional improvement. These approaches encompass lifestyle modifications, physical therapy, analgesics, nonsteroidal anti-inflammatory drugs (NSAIDs), and intra-articular corticosteroid injections [4]. Although these pharmacological treatments can provide symptomatic relief, they often fail to address the underlying pathology and may be associated with significant side effects affecting the gastrointestinal, cardiovascular, and renal systems [5]. For patients with advanced disease, surgical interventions such as total hip arthroplasty (THA) may be considered; however, surgery carries inherent risks and requires extensive rehabilitation.

In recent years, regenerative medicine has emerged as a novel therapeutic approach for musculoskeletal disorders, with platelet-rich plasma (PRP) injections gaining considerable attention. PRP is an autologous preparation of concentrated platelets in plasma, containing high levels of growth factors and bioactive molecules believed to promote tissue repair and modulate inflammation [6]. The rationale for using PRP in hip OA lies in its potential to enhance the body's intrinsic healing mechanisms, potentially slowing disease progression and improving clinical outcomes.

Despite the promising theoretical benefits of PRP therapy, its clinical application in hip OA has yielded mixed results, particularly regarding its efficacy in alleviating symptoms and improving joint function in comparison to other treatment modalities like HA intra-articular injections. Various studies, including systematic reviews and meta-analyses by Medina-Porqueres et al. and Veronesi et al. [7,8], have explored some of these outcomes. For instance, Medina-Porqueres et al. [7] examined four trials comparing HA to PRP outcomes in terms of pain or functional improvement. Two trials showed no difference between the groups while one trial showed better outcomes with PRP and the other showed better outcomes in favor of HA over PRP. Moreover, the safety profile of this intervention remains less well-defined, especially given the variability in PRP preparation protocols and the invasive nature of intra-articular injections.

Previous systematic reviews, such as the one by Belk et al. [9], have primarily focused on short-term efficacy of PRP in hip OA, without extensively addressing safety outcomes. By contrast, this review aims to provide a more comprehensive evaluation by assessing both long-term efficacy and safety. 

By systematically reviewing the available RCTs, we seek to provide a nuanced understanding of PRP therapy's efficacy and safety in hip OA, contributing to the growing body of literature on regenerative treatments and informing clinical decision-making. Furthermore, our findings will help identify gaps in current research and guide future investigations to optimize PRP protocols and enhance patient outcomes in hip OA management.

## Review

Methods

Search Strategy

Eligible articles were identified through searches of PubMed, Scopus, and the Virtual Health Library (VHL) databases up to October 31, 2022, by two independent reviewers. The terms "platelet-rich plasma", "PRP", "hip", and "femoroacetabular" were used in combination with Boolean operators (AND, OR) to find relevant articles. Medical Subject Heading (MeSH) terms were also utilized in PubMed to identify additional relevant articles. We followed the Preferred Reporting Items for Systematic Reviews and Meta-Analyses (PRISMA) guidelines [10].

Eligibility Criteria

Clinical studies evaluating PRP intra-articular injections into the hip joint as a treatment option for OA, regardless of grade, were included if the following criteria were met: (1) conducted as a randomized clinical trial; (2) included PRP as a treatment in at least one arm; (3) documented serious/severe adverse events and joint infections; (4) published within the last 10 years; and (5) published in English or Spanish. Studies were excluded if (1) PRP injections were not intra-articular; (2) studies were reviews, abstracts, surveys, letters, or editorials; (3) PRP preparation methods were not described; (4) plasma proteins/growth factors were used instead of PRP; and (5) articles were not accessible through the searched databases.

Data Extraction and Outcomes of Interest

Two investigators independently reviewed the included studies, extracting data into a predefined Excel spreadsheet with the following variables: (1, 2, 3) author, year, and title of study; (4) number of patients; (5) numbers of hips injected; (6) total number of PRP injections; (7) reported side effects; and (8) reported serious/severe adverse events. Outcomes of interest were any reported joint infection or serious/severe adverse events related to post-injection complications.

Methodological Quality Assessment

The methodological quality of the included studies was assessed using the Cochrane Risk of Bias Tool (RoB 2), which evaluates five main domains for possibility of bias, including bias arising from the randomization process (randomization and allocation concealment), bias due to deviations from intended interventions (blinding and protocol adherence) and missing outcome data (completeness of follow-up), bias in the measurement of the outcome (blinding of outcome assessors), and bias in the selection of the reported result (selective reporting).

Results

Study Selection

**Figure 1:**
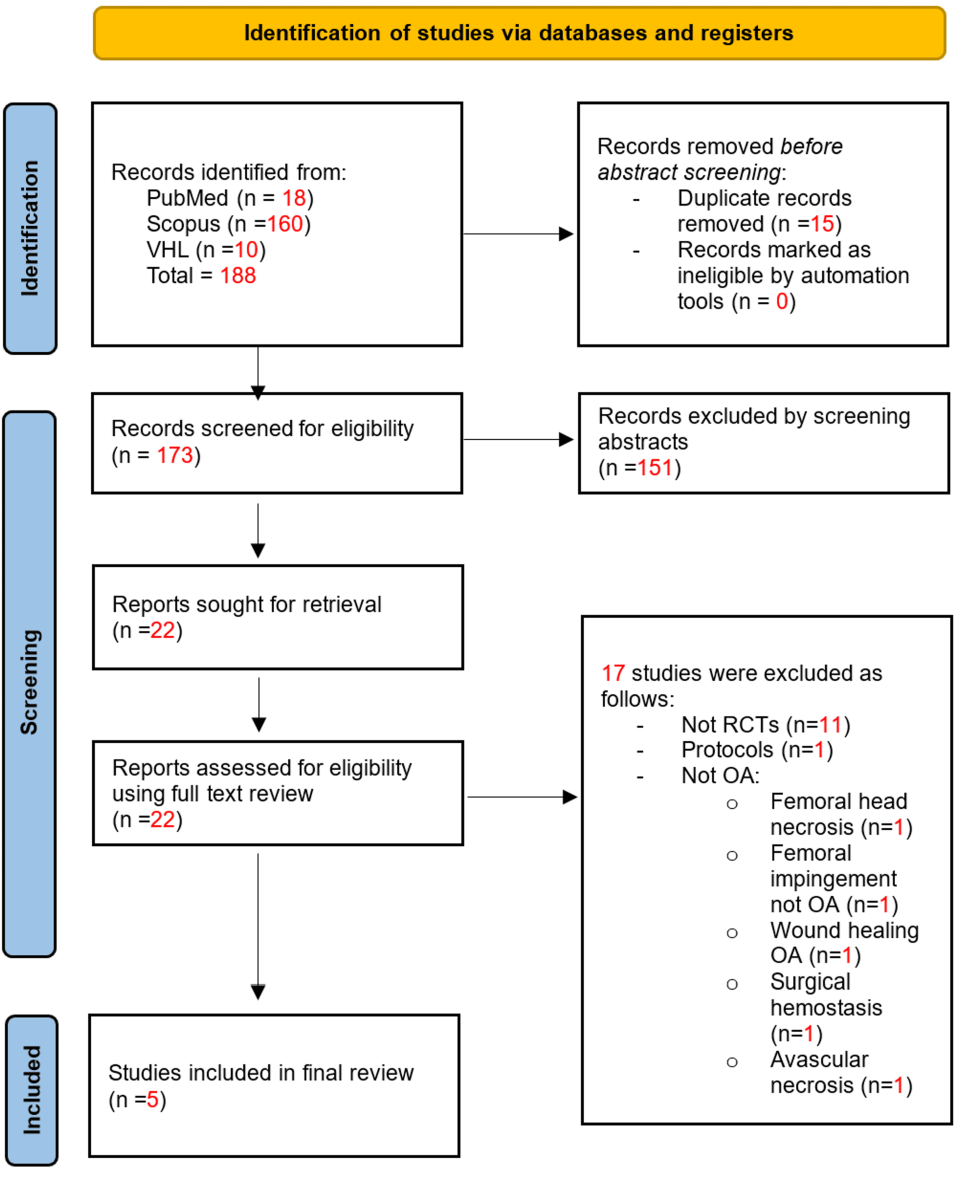
PRISMA flow diagram of the study selection process PRISMA: Preferred Reporting Items for Systematic Reviews and Meta-Analyses

The search yielded 188 studies (18 from PubMed, 160 from Scopus, and 10 from VHL). After removing 15 duplicates, 151 studies failed the abstract screening. Of the remaining 22 studies, 17 did not meet our criteria upon full-text review, resulting in five studies for inclusion [11-15]. Therefore, our systematic review synthesized data from five clinical trials comparing PRP injections to placebo or other modalities for the treatment of hip OA. Our process is outlined in the PRISMA flow diagram in Figure 1. A summary of the five included studies is provided in Table 1.

**Table 1:** Summary of the study characteristics and findings VAS: Visual Analog Score, HHS: Harris Hip Score, WOMAC: Western Ontario and McMaster Universities Osteoarthritis Index

Author	Year	Sample Size	Intervention	Control	Follow-up duration	Main outcomes		Safety findings	Quality assessment
						Pain (VAS)	Function		
Villanova-López et al. [11]	2020	74	PRP injections	Hyaluronic Acid (HA)	12 months	Significant pain reduction in the both groups. P-value < 0.01 at 12-month follow-up.	Significant improved function (HHS and WOMAC]. P-value < 0.01 at 12 months	No adverse effects were recorded (0 patients)	Low risk of bias
Di Sante et al. [12]	2016	43	PRP injections	Hyaluronic Acid	16 weeks	Significant pain reduction (VAS) at 4 weeks (p-value <0.01) but not at 16 weeks for PRP group (p-value > 0.05)	No significant improvement in function (WOMAC) at 4 or 16 weeks for PRP group (p-value > 0.05)	No complications (0 patients)	Some concerns
Dallari et al. [13]	2016	111	PRP injections	HA and combination therapy	12 months	Significant pain reduction (VAS) at all follow-up visits. PRP group had lowest VAS, especially at six months (p-value <0.0005 (PRP vs, HA) and p-value .007 (PRP vs. PRP-HA).	Significantly improved function (WOMAC), especially at 2 months (mean 73; 95% CI, 68-68) and 6 months (mean 72; 95% CI, 67-76) but not at 12 months (p-value > 0.05).	No serious adverse events	Low risk of bias
Nouri et al. [14]	2022	105	PRP injections	HA and combination therapy	6 months	Significant pain reduction (VAS) at all follow-up visits. p-value <01 all groups compared to the baseline.	Significantly improved function (WOMAC and Lequesne) at all follow-up visits. p-value <01 all groups compared to baseline. More significant functional improvement in PRP and PRP-HA groups compared to the HA group. P-value 0.041, 0.002, respectively)	Low rates of minor side effects (pain, warmth, stiffness) in 17 patients. PRP and PRP-HA groups had more pain compared to the HA group. P-value 0.001	Some concerns
Doria et al. [15]	2017	80	PRP injections	HA	12 months	Significant pain reduction in the both groups. P-value < 0.01 at six-month and 12-month follow-up.	Significant improved function (HHS and WOMAC). P-value < 0.01 at six months and 12 months	No major adverse events reported but significantly higher post-injection pain reaction in PRP group. P-value 0.043	Some concerns

Efficacy

PRP and pain: PRP injections consistently reduced pain in most patients for up to 12 months [11,13,14,15]. However, in the study by Dallari et al. [13], pain relief was short-lived and did not persist at follow-up at 16 weeks.

PRP and function: These clinical trials consistently utilized at least WOMAC to evaluate functional improvement [11,12,13,14,15]. There was consistent functional improvement with PRP injections at all follow-up visits in most trials [11,14,15]. However, in the study by Dallari et al. [13], no significant functional improvement was seen, and in the study by Dallari et al. [13], functional improvement was lost at 12 months of follow-up.

HA: HA injections also proved effective in managing hip OA. Significant pain reduction and functional improvement were reported [11,12,13,14,15]. However, it was outperformed by PRP in terms of pain reduction as reported by Dallari et al. [13] and functional improvement as reported by Nouri et al. [14].

Combination therapy: Combining PRP with HA did not yield significantly better outcomes compared to PRP alone, as noted in studies by Dallari et al. [13] and Nouri et al. [14]. This suggests that PRP may suffice for effective management.

Safety

Both PRP and HA demonstrated favorable safety profiles. No major adverse events were reported in any of the studies. Minor side effects, including localized pain or discomfort, were transient and resolved without additional treatment [11,12,13,14,15]. PRP-containing injections seem to cause more localized symptoms than HA alone as reported by Doria et al. [15].

Methodological Quality and Risk of Bias

We utilized the Cochrane Risk of Bias Tool (RoB 2) to assess the potential for bias, highlighting issues such as lack of describing randomization and blinding in some studies and differences in follow-up durations. Villanova-López et al. [11] and Dallari et al. [13] demonstrated solid methodological designs with an overall low risk of bias, while others, like Di Sante et al. [12], Nouri et al. [14], and Doria et al. [15], had issues such as unclear blinding. All studies, however, had robust outcome data with no missing data and no selective reporting. These differences may introduce bias and affect the reliability of the reported outcomes. Table 2 highlights our assessment of the included studies.

**Table 2:** Risk of Bias Assessment (RoB2) results 🟢: low risk, 🟡: some concerns, 🔴: high risk

Study	Randomization	Deviations from intended interventions	Missing outcome data	Outcome measurement	Selection of reported results	Overall risk
Villanova-López et al. [11]	🟢 Low: Adequately described randomization and allocation	🟢 Low: Double-blinding maintained	🟢 Low: No significant missing data	🟢 Low: Outcome assessors were blinded	🟢 Low: No selective reporting	🟢 Low
Di Sante et al. [12]	🟡 Some concerns: Randomization method not fully described	🟡 Some concerns: Blinding details insufficient	🟢 Low: Minimal missing data	🟡 Some concerns: Unclear maintenance of blinding of outcome assessors	🟢 Low: No selective reporting	🟡 Some concerns
Dallari et al. [13]	🟢 Low: Well-described with allocation concealment	🟢 Low: Double-blinding maintained	🟢 Low: Minimal missing data	🟢 Low: Blinded assessors and consistent outcome measurement	🟢 Low: Pre-specified outcomes reported	🟢 Low
Nouri et al. [14]	🟡 Some concerns: Inadequate description of allocation concealment	🟡 Some concerns: Blinding unclear across multiple groups	🟢 Low: Minimal missing data	🟡 Some concerns: Unclear blinding of outcome assessors	🟢 Low: No selective reporting	🟡 Some concerns
Doria et al. [15]	🟡 Some concerns: Allocation concealment not described	🟡 Some concerns: Blinding insufficient for providers	🟢 Low: Minimal missing data	🟡 Some concerns: Unclear blinding of assessors	🟢 Low: No selective reporting	🟡 Some concerns

Discussion

This systematic review evaluates the comparative effectiveness and safety of PRP versus placebo, hyaluronic acid (HA) injections, or HA-PRP combinations for managing hip OA. Our analysis of the five included studies reveals important insights into both efficacy and safety outcomes associated with PRP treatment.

Efficacy Comparison

Villanova-López et al. [11]: This study demonstrated significant pain reduction in both groups at 12 months post-injection (P < 0.01). In addition, significant improvements in function were observed based on the Harris Hip Score (HHS) and Western Ontario and McMaster Universities Osteoarthritis Index (WOMAC) (P < 0.01), confirming the long-term benefits of the treatment.

Di Sante et al. [12]: In this study, significant pain reduction was observed at four weeks for the PRP group (P < 0.01), but the effect did not persist at 16 weeks (P > 0.05). No significant improvement in function, as measured by WOMAC, was reported at either four or 16 weeks (P > 0.05), suggesting a limited functional impact of PRP in the short-term.

Dallari et al. [13]: This study reported significant pain reduction across all follow-up visits for the PRP group, with the greatest reduction observed at six months (P < 0.0005 vs. HA, P = 0.007 vs. PRP-HA). Functional improvements, measured by the WOMAC, were significant at two months (mean 73; 95% CI, 68-78) and six months (mean 72; 95% CI, 67-76), although these improvements were not maintained at 12 months (P > 0.05).

Nouri et al. [14]: Significant pain reduction (VAS) was observed in all treatment groups at all follow-up visits (P < 0.01 compared to baseline). Function, measured by the WOMAC and Lequesne indices, also improved significantly across all follow-up visits (P < 0.01 compared to the baseline). More substantial functional improvements were noted in the PRP and PRP-HA groups compared to the HA group (P = 0.041 and P = 0.002, respectively).

Doria et al. [15]: This randomized clinical trial showed significant pain reduction in both PRP and HA groups at six-month and 12-month follow-ups (P < 0.01). In addition, both HHS and WOMAC scores demonstrated significant functional improvements at both time points (P < 0.01), reinforcing the long-term efficacy of PRP and HA.

In summary, Villanova-López et al. and Doria et al. [11,15] highlighted significant pain and functional improvements at both six and 12 months. Di Sante et al. [12] found that PRP led to short-term pain reduction, but this did not translate into long-term pain relief or functional improvement. Dallari et al. [13] showed the strongest results at six months, with diminishing benefits at 12 months. Finally, Nouri et al. [14] suggested that combining PRP with HA does not significantly enhance outcomes, although PRP alone remains highly effective. Table 2 shows the efficacy comparison between the studies.

**Table 3:** Efficacy comparison HHS: Harris Hip Score, WOMAC: Western Ontario and McMaster Universities Osteoarthritis Index

Study	Pain reduction	Functional improvement
Villanova-López et al. [11]	Significant at 12 months (P < 0.01)	Significant at 12 months (HHS and WOMAC, P < 0.01)
Di Sante et al. [12]	Significant at 4 weeks (P < 0.01); No significance at 16 weeks (P > 0.05)	No significant improvement at 4 or 16 weeks (WOMAC, P > 0.05)
Dallari et al. [13]	Significant at all follow-ups, greatest at 6 months (P < 0.0005)	Significant at 2 and 6 months (WOMAC); Not significant at 12 months (P > 0.05)
Nouri et al. [14]	Significant at all follow-ups (P < 0.01)	Significant at all follow-ups (WOMAC and Lequesne, P < 0.01)
Doria et al. [15]	Significant at 6 and 12 months (P < 0.01)	Significant at 6 and 12 months (HHS and WOMAC, P < 0.01)

Safety Comparison

Villanova-López et al. [11]: This study included 74 patients and reported no adverse effects in either the PRP or HA groups, indicating a strong safety profile for PRP injections.

Di Sante et al. [12]: In a cohort of 43 patients, the study found no complications in either the PRP or HA treatment groups, reinforcing the safety of both therapies.

Dallari et al. [13]: With 111 participants, this study reported no serious adverse events associated with PRP injections, further supporting their safety.

Nouri et al. [14]: Among 105 patients, minor side effects, such as pain, warmth, and stiffness, were observed in 17 individuals. The PRP and PRP-HA groups experienced more pain compared to the HA group (P-value 0.001), but the overall safety remained high.

Doria et al. [15]: This study involved 80 patients and reported no major adverse events, although a significantly higher post-injection pain reaction was noted in the PRP group compared to the HA group (P-value 0.043).

Overall summary

Across all five studies, PRP injections were found to be well-tolerated, with minimal adverse events reported. No major complications were associated with either PRP or HA treatments, reinforcing the safety profile of PRP as a therapeutic option. Table 3 shows a summary of the safety comparison in the reported studies.

**Table 4:** Safety comparison PRP: platelet-rich plasma, PRP-HA: platelet-rich plasma/hyaluronic acid

Study	Sample size	Adverse events
Villanova-López et al. [11]	74	No adverse effects reported (0 patients)
Di Sante et al. [12]	43	No complications reported (0 patients)
Dallari et al. [13]	111	No serious adverse events reported
Nouri et al. [14]	105	Minor side effects (pain, warmth, stiffness) in 17 patients; more pain in the PRP and PRP-HA groups (P-value 0.001)
Doria et al. [15]	80	No major adverse events; significantly higher post-injection pain reaction in the PRP group (P-value 0.043)

Limitations and variability

Study Design Variability

The studies exhibited significant variability in terms of PRP preparation methods, HA formulations, and follow-up durations. This variability complicates the direct comparison of results and emphasizes the need for standardized protocols. For instance, differences in PRP concentration, preparation, and processing techniques may impact both efficacy and safety outcomes. The diverse patient populations, along with methodological differences, further complicate the generalizability of the findings across studies.

Future Research Directions

Standardization of protocols: Future research should aim to standardize PRP preparation and administration protocols, ensuring more reliable comparisons across studies.

Personalized treatment strategies: Identifying patient-specific factors, such as age, disease stage, or comorbidities, that predict response to PRP versus HA could improve treatment personalization.

Long-term studies: Extended follow-up studies are necessary to assess the long-term sustainability of PRP’s therapeutic benefits and to explore its role as a part of a broader management strategy for hip OA.

## Conclusions

The comparative analysis of efficacy and safety from these studies supports the use of PRP as a promising treatment for hip OA. While HA also provides effective symptom relief, PRP may offer significant advantages, especially for patients who do not respond to conventional treatments. Findings from clinical trials highlight PRP’s potential for meaningful clinical improvement while being a safe treatment modality. Future research should focus on standardizing PRP protocols and assessing its long-term safety and efficacy across diverse patient populations.
